# A Facile Microwave-Promoted Formation of Highly Photoresponsive Au-Decorated TiO_2_ Nanorods for the Enhanced Photo-Degradation of Methylene Blue

**DOI:** 10.3390/nano14221780

**Published:** 2024-11-05

**Authors:** Andreea Bondarev, Sonia Mihai, Abubakar Katsina Usman, Diana Luciana Cursaru, Dănuţa Matei, Veronica Sătulu, Cătălina Gheorghe, Gheorghe Brănoiu, Raluca Şomoghi

**Affiliations:** 1 Faculty of Petroleum Refining and Petrochemistry, Petroleum—Gas University of Ploiesti, 100680 Ploiesti, Romania; andreeabondarev22@gmail.com (A.B.); dianapetre@upg-ploiesti.ro (D.L.C.); danuta.matei@upg-ploiesti.ro (D.M.); catalina.gheorghe@upg-ploiesti.ro (C.G.); gbranoiu@yahoo.com (G.B.); r.somoghi@gmail.com (R.Ş.); 2 Department of Pure and Industrial Chemistry, Bayero University, Kano PMB 3011, Nigeria; aukatsina.chm@buk.edu.ng; 3 National Institute for Laser, Plasma and Radiation Physics, 409 Atomistilor Str., 077125 Magurele, Romania; veronica.satulu@inflpr.ro; 4 National Institute for Research and Development in Chemistry and Petrochemistry, Spl. Independentei, 060021 Bucharest, Romania

**Keywords:** photodegradation, methylene blue, nanorods, microwave synthesis, Au nanoparticles

## Abstract

The integration of noble metal nanoparticles (NPs) effectively modifies the electronic properties of semiconductor photocatalysts, leading to improved charge separation and enhanced photocatalytic performance. TiO_2_ nanorods decorated with Au NPs were successfully synthesized using a cost-effective, rapid microwave-assisted method in H_2_O_2_ and HF media for methylene blue (MB) degradation under visible light illumination. X-ray diffraction (XRD), X-ray photoelectron spectroscopy (XPS), scanning electron microscopy (SEM), transmission electron microscopy (TEM), N2 physisorption, and UV–vis spectroscopy were employed to characterize the structures, morphologies, compositions, and photoelectronic properties of the as-synthesized materials. The fusing of Au NPs effectively alters the electronic structure of TiO_2_, enhancing the charge separation efficiency and improved electrical conductivity. The HF treatment promotes the exposure of the highly reactive (001) and (101) crystalline facets. The improved photocatalytic activity of Au/TiO_2_, achieving 97% efficiency, is attributed to the surface plasmon resonance (SPR) effect of the Au NPs and the presence of oxygen vacancies. The photodegradation of MB using the TiO_2_/Au photocatalysts follows pseudo-first-order kinetics, highlighting the enhanced catalytic efficiency of the synthesized nanostructures. The exceptional properties of the binary Au/TiO_2_ photocatalysts, including the SPR effect, exposed crystallographic faces, and efficient charge carrier separation through a decrease in the recombination of electrons and holes, contribute to the photocatalytic degradation of MB.

## 1. Introduction

Environmental pollution caused by rapid industrialization poses a colossal threat to human health, tropical rainforests, and the wider environment [1]. Pollution, including water, air, soil, and waste perturbs the ecosystem and can lead to respiratory diseases, cardiovascular diseases, asthma, and other health issues [2]. Water pollution is particularly of great concern because all other forms of pollution eventually make their way to water. Water pollution has deleterious impacts on the environment, leading to environmental degradation and riverbank erosion [3]. It also affects water quality, accessibility, and sustainability, which are essential for the well-being of both humans and the environment [4]. Industrial organic dyes such as MB, Rose bengal (RB), eosin Y (EY), Rhodamine B (RhB), etc., are major contributors to water pollution. These dyes are extensively employed in various industries such as textile, paper, cosmetic, pharmaceutical, and food, leading to severe environmental toxicity, particularly in water bodies [5]. The discharge of textile wastewater alone accounts for approximately 15% of the global production of dyes, with the majority of dye wastewater generated by the food, chemical, and textile industries [6]. These dyes are toxic, mutagenic, and carcinogenic, posing a threat to both the environment and human health and their presence in water blocks sunlight, inhibiting the photosynthesis process of aquatic plants [7,8].

Various methods have been explored to treat dye wastewater, including biological, physical, and chemical processes [9]. Moreover, photocatalytic water treatment technology has shown auspicious outcomes in the remediation of toxic organic dyes and microorganisms from water sources with faster reaction kinetics, enhanced pollutant degradation, and a reduction in toxicity [10,11]. It is considered an advanced oxidation process (AOP) and is particularly effective in treating non-biodegradable, toxic, and recalcitrant pollutants that are resilient to conventional treatment methods [12]. Photo-assisted catalysis plays a significant role in wastewater treatment by utilizing a semiconductor activated by a light source to generate hydroxyl radicals, which can mineralize organic compounds and degrade pollutants [13]. TiO_2_ semiconductor is a promising material for various photocatalytic applications due to its excellent stability, non-toxicity, and cost-effectiveness [14]. It is commonly used as a photocatalyst for single electron transfer in a range of applications, such as the degradation of dyes and surfactants in wastewater treatment [15].

The photocatalytic activity of TiO_2_ is influenced by factors such as the chemical potential of electrons, charge transport properties, band gap energy, and concentration of surface-active sites [16]. Despite being extensively studied, there are still uncertainties and limitations regarding the TiO_2_-based photocatalytic systems. Its wide bandgap limits its ability to absorb a significant portion of the solar spectrum [17,18], which restricts its efficiency in solar energy conversion and storage [19]. Uncertainties include the pathways involving visible light activity, charge transfer between semiconductors and metal nanoparticles, and factors determining selectivity [20].

The effectiveness of metal oxide photocatalysts for photocatalytic applications, such as TiO_2_, ZnO, WO_3_, etc., can be boosted via band engineering [21], morphology control, and defect engineering techniques. One approach is doping metal oxides with noble metals such as precious metals or transition metals to enhance their catalytic efficiency and extend their absorption range to visible light [22]. Noble metal doping introduces additional active sites and modifies the electronic configuration of the catalysts, leading to improved charge separation and increased electrical conductivity [23]. The presence of defects on grain boundaries, oxygen vacancies, and heterojunctions also plays a vital role in enhancing the performance of metal oxide photocatalysts [24]. Other methods involve ion doping, surface defect engineering, sensitization, morphology control, and the modulation of an inner electric field through band engineering techniques, which promotes photocarrier separation and transportation [25]. For example, Hernandez et al. [26] investigated the doped Cu, Ag, and Eu in nanostructured TiO_2_ to determine the location of metal ions, their stable valence states, and their impact on the structural, electronic, and optical properties of TiO_2_. One important finding was that the metal-ion doping of TiO_2_ can result in a lower anatase to rutile phase transformation temperature and a redshift of the photophysical response, leading to enhanced visible light photoreactivity. Similarly, Huang et al. employed different noble metals (Au, Ag, Cu, Pt, Pd) to modify TiO_2_ hierarchically structured microspheres, and the experimental results show that the photocatalytic activities of the noble metal-modified microspheres are significantly enhanced, particularly for the Pt-modified sample [27]. TiO_2_ synthesis has been studied extensively in the literature using different classical approaches from chemical, physical, and biological angles, such as the following: sol–gel, hydrothermal, thermal decomposition, laser irradiation, electrolysis and so on, but there are fewer studies on microwave-assisted synthesis [28].

This study presents a novel approach for the synthesis of TiO_2_ and Au/TiO_2_ nanostructures via a cost-effective and rapid microwave-assisted method. The prepared nanostructures were successfully tested in the photocatalytic degradation of MB under simulated visible light. The structural characteristics of the nanostructures are significantly influenced by the reaction medium, consisting of H_2_O_2_ and HF, promoting the exposure of highly reactive (001) and (101) crystalline facets. Through analyzing the experimental results, we have proposed a possible reaction mechanism.

## 2. Materials and Methods

### 2.1. Materials and Reagents

Analytical grade titania, hydrogen fluoride, hydrogen peroxide (Fluka Chemicals, Buchs, Switzerland), and Tris(triphenylphosphine gold)oxonium tetrafluoroborate {[O(Au PPh_3_)_3_][BF_4_]; (Burlington Chemical, Inc., Burlington, MA, USA) were employed without further treatment. Double distilled water (DDI) was used as a solvent throughout the research unless otherwise stated.

### 2.2. Microwave Synthesis of TiO_2_ and Au/TiO_2_

Yellow titanium oxide (TiO_2_) powder with peroxide groups was successfully prepared using microwave fields. A total of 3 g of TiO_2_ powder was mixed with 6 mL of HF (47%), 10 mL of H_2_O (DDI), and 10 mL of H_2_O_2_ 30%. Afterwards, the solution was put in the microwave oven and heated to 700 W for 28 min at 180 degrees Celsius. After cooling to room temperature and precipitating with NH_4_OH 25%, the solution was filtered and calcined for 3 h at 450 degrees Celsius. Au/TiO_2_ nanorods were synthesized following the same procedure, with the addition of 3.19 weight % Au NPs before the calcination. The microwave synthesis procedure was conducted in triplicate under identical conditions to ensure the reproducibility of the process. The particle size distribution for the three batches of synthesized photocatalysts (labeled L1, L2, and L3) was measured and the statistical analysis and particle distribution data are presented in Table 1 below.

### 2.3. Characterization

The morphology and elemental composition of the photocatalysts were analyzed using a FEI Tecnai G2 F-20 TwinCryo High Resolution Microscope (HR–TEM) (FEI American Company, Brno, Czech Republic), operated at an acceleration voltage of 200 KV with a magnification of 80,000 to 20,000 and field scanning electron microscopy FE-SEM Scios 2 Hivac Dual-Beam (FEI American Company, Brno, Czech Republic) with energy dispersive X-ray spectroscopy. The UV-Vis spectra were analyzed using a UV-Vis spectrophotometer Shimatzu 2600 UV-Vis NIR. The chemical composition of the photocatalysts was investigated using X-ray Photoelectron Spectroscopy (XPS) with a K-Alpha Thermo Scientific spectrometer (ESCALAB™ XI+, East Grinstead, UK) equipped with a 180° double-focusing hemispherical analyzer. The peak positions were calibrated against the adventitious C1s peak at 284.8 eV, as specified by the Avantage data software (Thermo Avantage v5.9921, East Grinstead, UK). The surface elemental composition was determined by recording survey spectra at a pass energy of 50 eV. The elemental bonding states of the Au/TiO_2_ photocatalysts were further assessed by acquiring high-resolution spectra for the binding energy regions of C1s, O1s, Ti 2p, and Au 4f at a pass energy of 20 eV. Data acquisition and spectra processing were carried out using the aforementioned Avantage software. The XRD pattern was recorded in the 2 theta measurement range between 20–80° at a 5°/min scan rate employing a Bruker D8 Advance diffractometer (Karlsruhe, Germany; θ-θ type, Cu-Kα radiation (λ = 1.5418 Å), 40 kV, and 40 mA).

For the BET surface and pore size distribution, the photocatalyst samples were outgassed in vacuum at 300 °C for 3 h prior to the physisorption analysis. The specific surface area was determined by the Brunauer–Emmett–Teller (BET) method in a pressure range of 0.045 ≤ p/p◦ ≤ 0.25, while the pore size distributions were calculated with the density functional theory (DFT) model.

### 2.4. Photocatalytic Activity Tests

The photocatalytic activity of the TiO_2_ and Au/TiO_2_ nanorods was evaluated by the mineralization of MB solution. A total of 0.01 g of photocatalyst was mixed with 50 mL of 32 mg/L MB solution for 60 min in the dark to establish the adsorption–desorption equilibrium between the dye solution and photocatalyst. After equilibrium (60 min), 2 mL of the solution was collected and the initial absorbance was measured. The mineralization reaction was carried out using a Toption-type photoreactor equipped with a Xe lamp with λ > 400 nm at an ambient temperature under continuous stirring. A UV-vis spectrophotometer was utilized to monitor the reaction, measuring the absorbance of 665 nm every 10 min.

## 3. Results and Discussion

### 3.1. XRD Analysis

**Figure 1:**
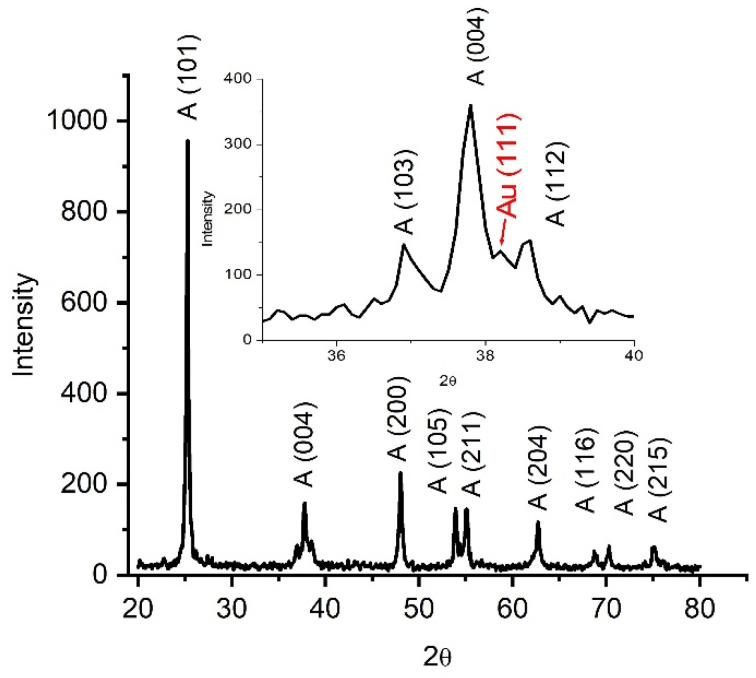
Powder XRD patterns of Au/TiO_2_ nanorods.

The crystalline structures of the photocatalysts were characterized by XRD. Figure 1 shows the characteristic peaks of the TiO_2_ anatase phase. The diffraction peaks at the 25.3, 37.7, 48, 53.9, 55.2, 62.7, 68.65, 70.2, and 74.94° 2θ values correspond to the (101), (004), (200), (105), (211), (204), (116), (220), and (215) crystal planes. The quantitative analysis of Au/TiO_2_ by the Rietveld method revealed a tetragonal structure belonging to the symmetry group I 41/AMD with a = b = 3.7892Å and c = 9.5154 Å, a crystallite size Lorentzian at 17.4 nm for TiO_2_, and a symmetry group Fm-3m with a crystallite size Lorenzian 15.6 nm with lattice parameters a = b = c = 4.08 Å for gold. The interplanar distance for the TiO_2_ (101) plane d = 3.52 Å and d = 2.35 Å for gold plane (111), respectively, which were determined and were in agreement with the distance determined by FFT (Fast Fourier Transform)—TEM. The peak corresponding to Au NPs can be observed at a 2θ value of 38.1°.

### 3.2. XPS Analysis

**Figure 2:**
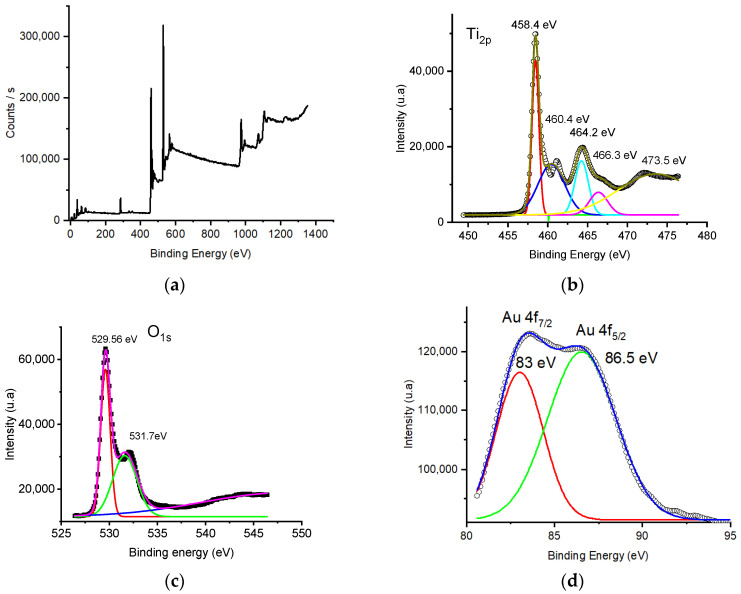
XPS spectra: survey spectrum (**a**); deconvoluted Ti 2p spectra (**b**); deconvoluted O 1s spectra (**c**); deconvoluted Au 4f spectra (**d**).

Figure 2 presents a detailed analysis of the chemical bonding mechanisms in Au/TiO_2_ nanorods, derived from extensive X-ray photoelectron spectroscopy (XPS) investigations. It offers valuable information on surface elemental composition and specific binding energy values, facilitating a deeper understanding of chemical states and interactions. Based on the XPS survey spectra shown in Figure 2a, it is clear that the as-obtained Au/TiO_2_ nanorods primarily consist of titanium, gold, oxygen, and carbon. The presence of the carbon peak in the survey spectra is attributed to both the carbon strip on which the sample was deposited and the incidental carbon resulting from sample exposure to the ambient environment. Figure 2b–d show the high-resolution spectra of the Ti2p, Au4f, and O1s regions. The Ti2p spectra reveal that titanium exhibits two distinct doublets with a splitting energy of ΔE = 5.8 eV. Specifically, titanium dioxide, TiO_2_ (Ti^4+^), is characterized by peaks at 458.4 eV and 464.2 eV, constituting 61% of the composition, while dititanium trioxide, Ti_2_O_3_ (Ti^3+^), is represented by peaks at 460.4 eV and 466.3 eV, accounting for 39% of the composition. The further analysis of the O1s high-resolution spectrum reveals two distinct peaks with binding energies at 529.56 eV and 531.7 eV, corresponding to the Ti–O bond and the presence of hydroxyl groups and oxygen vacancies (O_V_), respectively [29,30,31].

After analyzing the wide peak in the 80–90 eV range, it was identified a distinct doublet with peaks at binding energies of 83 eV and 86.5 eV. These peaks correspond to the Au 4f7/2 and Au 4f5/2 orbitals of gold nanoparticles, respectively. The measured splitting energy (ΔE) between these two states is 3.5 eV, which is consistent with the typical energy separation.

### 3.3. Morphological Studies

**Figure 3:**
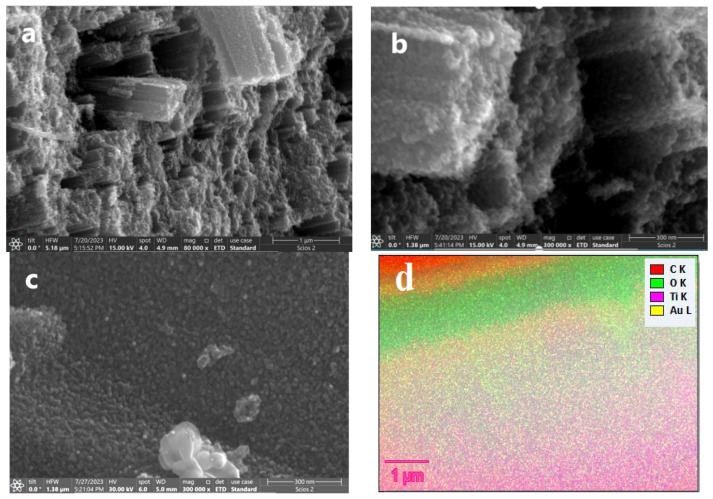
SEM images for yellow TiO_2_ (nanorods) (**a**) and (**b**); Au/TiO_2_ (**c**); EDS map for Au/TiO_2_ (**d**).

**Figure 4:**
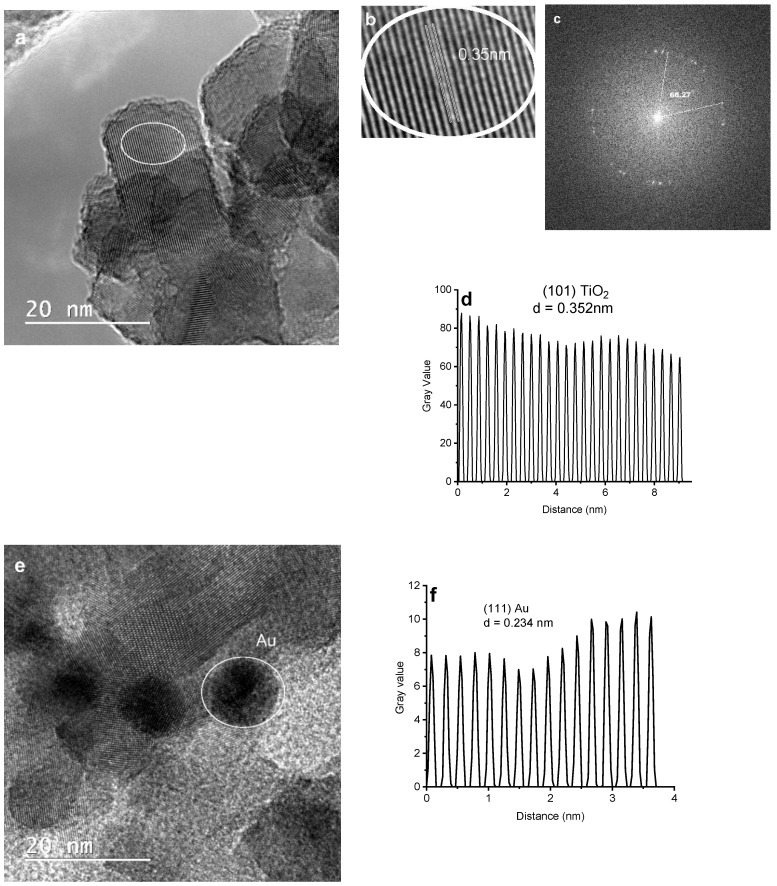
HR-TEM image: TiO_2_ nanosheets (**a**,**b**); the corresponding FFT pattern (**c**); magnified lattice fringes for d-spacing calculation (101) (**d**); Au/TiO_2_ (**e**); magnified lattice fringes for d-spacing calculation (111) Au (**f**).

Scanning electron microscopy (SEM) and transmission electron microscopy (TEM) were employed to analyze the microstructural morphologies of the samples. Figure 3a,b illustrate the SEM micro-images of the yellow TiO_2_ nanorod clusters, measuring 80 μm in length and 50–80 nm in width, formed through the assembly of TiO_2_ nanosheets with dimensions around 10–20 nm and an interplanar spacing of d = 0.352 nm assigned to the (101) plane according to XRD. The SEM image of Au-doped TiO_2_ in Figure 3c revealed that the Au NPs with an average diameter of about 10–15 nm have been uniformly distributed onto the surface of the TiO_2_ nanorods in contrast to the SEM image of the yellow TiO_2_. Figure 3d shows the elemental EDS analysis, further confirming the presence of Au NPs in the Au/TiO_2_ photocatalysts. Figure 4a,b show high-resolution TEM (HR-TEM) images taken from a single nanosheet TiO_2_ and Au-doped TiO_2_ nanorod, with the darker particles randomly distributed on the TiO_2_ surface assigned to the Au-NPs in Figure 4e. Figure 4d depicts the face of Au/TiO_2_, representing the noise-refined legible TEM lattice fringes with a perpendicular distance of 0.234 nm, corresponding to the interplanar spacing of d = 0.235 nm for FCC Au (111) planes [32]. As shown in the figure FTT (Fast Fourier Transform) pattern in Figure 4c, the angle at 68.27° is assigned to the theoretical values of the angle between the planes (001) and (101) [33]. The HRTEM images in Figure 4a show some TiO_2_ particles, as does Figure 4b.

### 3.4. N_2_ Physisorption

**Figure 5:**
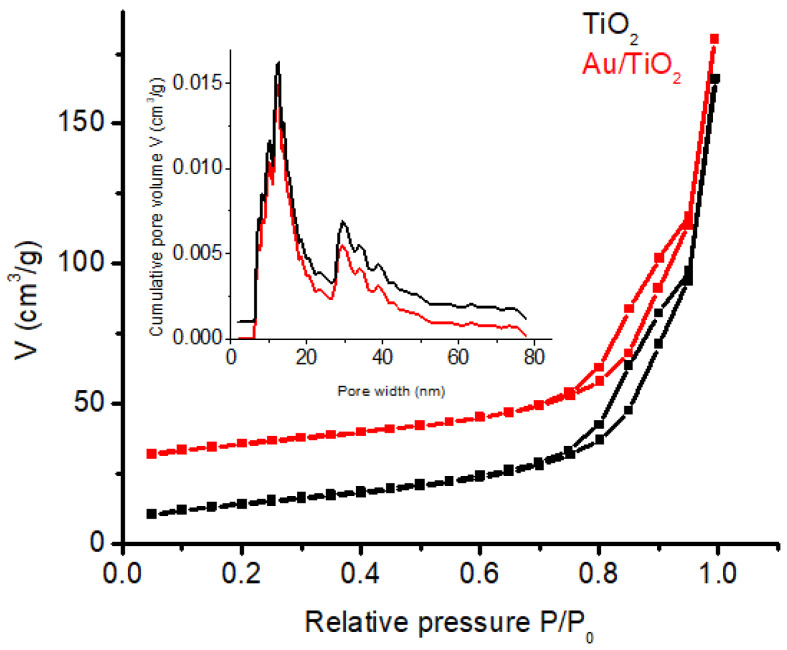
Nitrogen adsorption—desorption isotherms for yellow TiO_2_ (black line) and Au/TiO_2_ (red line). The detail represents the pore distribution for the TiO_2_ (black line) and Au/TiO_2_ (red line).

As illustrated in Figure 5, the adsorption–desorption isotherms for both the Au/TiO_2_ and yellow TiO_2_ samples exhibited identical type IV isotherms with H3 hysteresis loops around 0.75 < P/P_0_ < 0.95 according to the IUPAC classification [34], indicating the development of a mesoporous structure in both samples. The BET surface areas for the two samples were calculated as follows: 50.83 m^2^g^−1^ for TiO_2_ and 50.13 m^2^g^−1^ for Au/TiO_2_. The inset in Figure 5 represents the DFT pore size distribution of the samples which is centered mainly around 12.55 nm, with a total pore volume of 0.227 cm^3^/g for TiO_2_ and 0.219 cm^3^/g for Au/TiO_2_. Doping with Au NPs did not significantly influence the specific surface area of the catalyst.

### 3.5. Optical Studies

**Figure 6:**
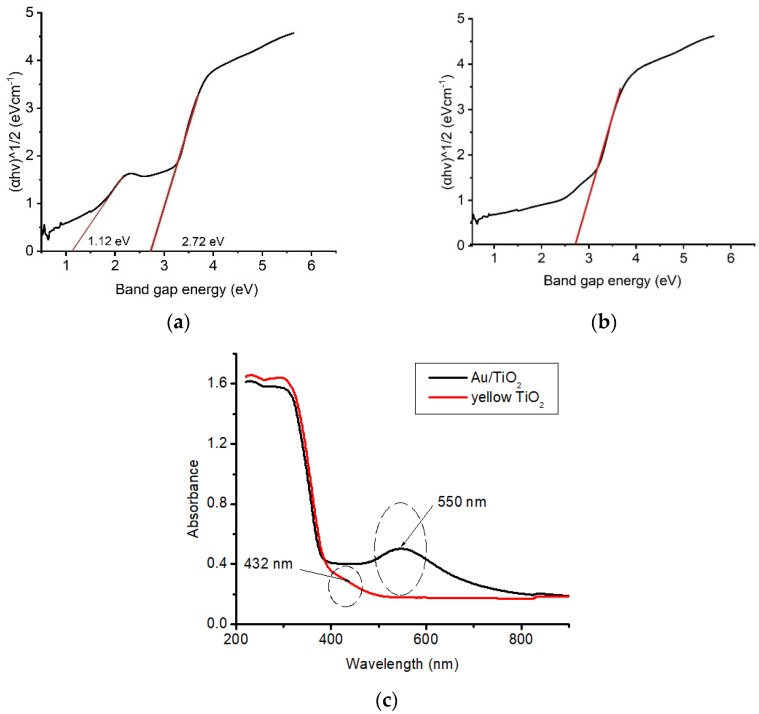
Determination of band gap energy for Au/TiO_2_ (**a**); yellow TiO_2_ (**b**); UV-Vis absorption spectrum (**c**).

The diffusion spectra of the yellow TiO_2_ and Au//TiO_2_ photocatalysts are presented in Figure 6. An absorption band at 400 nm is observed for yellow TiO_2_, while the Au/TiO_2_ photocatalyst shows an absorption band around 550 nm, which could be attributed to the fused Au NPs in the Au/TiO_2_ photocatalyst. Using the Tauc equations, it was discovered that the Au/TiO_2_ case saw a significant decrease in the band gap from 3.2 eV to 1.12 eV for pure TiO_2_. Yellow TiO_2_ has a lower band gap energy of 2.72 eV than 3.2 eV, enough to allow it to access the visible light region during the photodegradation process, improving the separation of charge carriers.

### 3.6. Photocatalytic Activity

**Figure 7:**
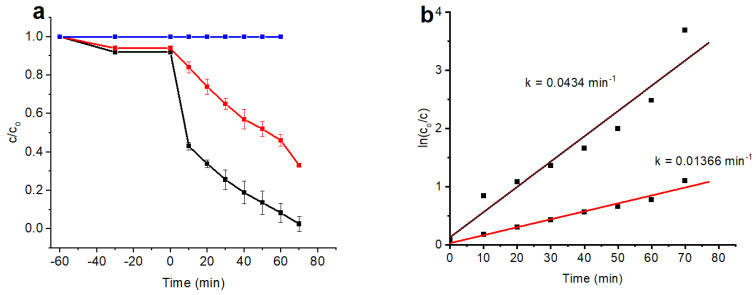
Photodegradation efficiency of MB: without photocatalyst (blue line); yellow TiO_2_ (red line); Au/TiO_2_ (black line) (**a**); the pseudo-first-order kinetic rate plot (**b**).

**Figure 8:**
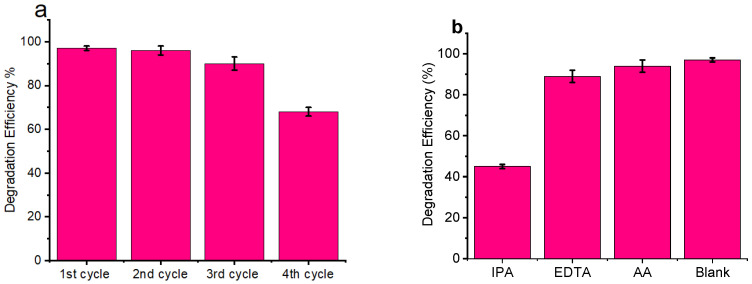
Photocatalytic stability (**a**); scavenger test (**b**).

The degradation of MB dye was evaluated by simulated visible light irradiation with Au/TiO_2_ nanorods and yellow TiO_2_. The samples were subjected to visible light irradiation for 70 min after the adsorption–desorption equilibrium was established. After establishing the adsorption–desorption equilibrium, the samples were exposed to visible light irradiation for 70 min. Figure 7a shows the time-dependent changes in the concentration of the dye, with Au/TiO_2_ achieving a high degradation efficiency of 49% after the first 10 min compared to just 7% for yellow undoped TiO_2_. After 70 min, the degradation efficiency of the Au/TiO_2_ reached 97%, outperforming the undoped yellow TiO_2_ catalyst at only 66%. Figure 7b presents the degradation kinetics of the samples based on pseudo-first-order plot. Au/TiO_2_ exhibited an initially high degradation rate, seven times faster than that of yellow TiO_2_ during the first 10 min, which then plateaued, maintaining a constant rate from the 10th to the 70th minute. The TiO_2, however,_ maintained a constant rate throughout, indicative of the pseudo-first-order kinetics being present from the beginning. This difference suggests that the degradation rate of Au/TiO_2_ is more concentration-dependent in the early stages compared to that of yellow TiO_2_. The improved performance of Au/TiO_2_ can be attributed to the visible light extension, the formation of massively reactive oxidizing species, and an enhanced charge transfer mechanism. The evaluation of the photocatalytic stability and reusability of Au/TiO_2_ was estimated according to the same procedure of photodegradation, performing recycling investigations over four degradation cycles successively, as shown in Figure 8a.

### 3.7. Scavenger Test

To determine the active species in the degradation of MB using Au/TiO_2_ nanorods, different radical scavengers, including isopropyl alcohol (IPA), ascorbic acid (AA), and ethylenediaminetetraacetic acid (EDTA) were employed in degradation experiments to trap hydroxyl radicals (•OH), superoxide radicals (•O_2_^−^), and photogenerated holes (h^+^) [35,36]. The reaction performance is illustrated in Figure 8b. Among the three scavengers, IPA had the most pronounced effect on MB degradation under visible light, causing a significant reduction in efficiency, with EDTA and AA showing no obvious effects. This indicates that the •OH radicals play a vital role in the degradation process of MB over Au/TiO_2_ nanorods [37].

### 3.8. Proposed Mechanism

The reaction mechanism proposed, based on the photocatalytic reaction data, is outlined by Equations (1)–(4) below [38,39,40]. In essence, photo-excited charge carriers are produced upon the absorption of light by the as-synthesized Au/TiO_2_ nanorods. The enhanced absorption in the visible light range is attributed to the dispersed Au NPs on the surface of TiO_2_. The photo-generated holes in the valence band (VB) of TiO_2_ migrate to the surface of the catalyst, where they react with adsorbed hydroxyl ions (${\mathrm{OH}}_{\mathrm{ads}}^{-}$) to form highly reactive hydroxyl radicals (•OH). Simultaneously, the electrons that are excited into the conduction band (CB) of the catalyst reduce the amount of oxygen (O_2_) molecules, resulting in the formation of superoxide radicals ($•O_{2}^{-}$), as shown in the following equations:

(1) $$\mathrm{Au}/{\mathrm{TiO}}_{2}\overset{\mathrm{hv}\;}{\Rightarrow }\mathrm{Au}/{\mathrm{TiO}}_{2}\left(e_{\mathrm{CB}}^{-}+h_{\mathrm{VB}}^{+}\right)$$

(2) $${\mathrm{OH}}_{\mathrm{adsorbed}}^{-}+h_{\mathrm{VB}}^{+}\to •\mathrm{OH}\;\mathrm{radicals}$$

(3) $$O_{2}+e_{\mathrm{CB}}^{-}\to •O_{2}^{-}$$

(4) $$\mathrm{Au}/{\mathrm{TiO}}_{2}\;\left(•\mathrm{OH}/•O_{2}^{-}\right)+\mathrm{MB}\;\mathrm{dye}\;\to {\mathrm{CO}}_{2}+H_{2}O$$

The highly oxidized ${•\mathrm{OH}\;\mathrm{and}\;•O}_{2}^{-}$ radicals produced in (2) and (3) above can both oxidize the organic MB dye coming into contact with the catalyst surface. Equation (4) below represents the degradation reaction of MB dye with •OH or $•O_{2}^{-}$ radicals. The photo-oxidation reactions pass through reaction intermediates that form CO_2_ and H_2_O on further oxidation reaction. However, the scavenger test results indicate that $•O_{2}^{-}$ radicals are the dominant reactive species, as their inhibition significantly reduced the photocatalytic activity of Au/TiO_2_ nanorods against the dye molecules, suggesting their crucial role in driving the overall reaction mechanism (Equation (5)).

(5) $$\mathrm{Au}/{\mathrm{TiO}}_{2}\;\left(•\mathrm{OH}\right)+\mathrm{MB}\;\mathrm{dye}\;\to \mathrm{intermediates}+{\mathrm{CO}}_{2}+H_{2}O$$

## 4. Conclusions

The synthesized Au-decorated TiO_2_ and yellow TiO_2_ nanorods were successfully produced by a microwave-assisted method, resulting in nanosheets with exposed (001) facets. The photocatalytic performance was considerably boosted by the synergistic effect between the nanostructure’s shape, size, and Au NPs doping. The synergistic effect of the nanostructure’s shape, size, and Au NPs doping significantly enhanced photocatalytic performance. The photocatalysts exhibited excellent efficiency in degrading MB under visible light, with the Au/TiO_2_ system achieving 97% photodegradation due to the surface plasmon resonance effect, effective charge separation, and the exposure of reactive crystalline facets. These findings point out the potential of nanostructures based on TiO_2_-for photocatalytic applications.

## Figures and Tables

**Table 1:** Particle size distribution for the 3 batches of the as-synthesized catalysts.

Variable	Total Count	Mean	SE Mean	StDev	Minimum	Maximum
L1	101	14.712	0.325	3.249	5.595	24.060
L2	105	14.368	0.257	2.620	8.986	22.318
L3	80	14.997	0.320	2.843	10.025	23.288

## Data Availability

Data will be made available upon request.
